# Analysis of the correlation between cervical HPV infection, cervical lesions and vaginal microecology

**DOI:** 10.3389/fcimb.2024.1405789

**Published:** 2024-08-16

**Authors:** Zhongru Fan, Dongyu Han, Xin Fan, Yu Zeng, Lin Zhao

**Affiliations:** ^1^ Department of Urology, The Affiliated Suzhou Hospital of Nanjing Medical University, Suzhou Municipal Hospital, Gusu School, Nanjing Medical University, Suzhou, China; ^2^ Department of Obstetrics and Gynecology, Suzhou Hospital, Affiliated Hospital of Medical School, Nanjing University, Suzhou, China; ^3^ Department of Obstetrics and Gynecology, The Second Hospital of Dalian Medical University, Dalian, China; ^4^ Department of Obstetrics and Gynecology, Chengdu Women’s and Children’s Central Hospital, School of Medicine, University of Electronic Science and Technology of China, Chengdu, China

**Keywords:** HPV infection, cervical intraepithelial neoplasia, cervical cancer, vaginal microecology, 16srRNA

## Abstract

**Background:**

Vaginal microbiota is involved in human papillomavirus (HPV) infection and cervical cancer (CC) progression, and the specific changes in vaginal microbial composition during this process remains uncertain.

**Objective:**

This study aimed to observe the changes in the specific composition of vaginal microorganisms in different cervical lesions and identify biomarkers at different stages of lesions.

**Methods:**

In this study we used the illumina high-throughput gene sequencing technology to determine the V4 region of 16SrRNA and observed the vaginal microbial composition in different cervical lesions.

**Results:**

The vaginal microbiota of patients with high-risk HPV infection and cervical lesions is significantly different from that of the normal population, but there is no significant difference in the richness of vaginal microbes. The diversity of vaginal species in CC patients is higher than that in high-risk HPV infection or CIN patients. The main manifestation is an increase in the diversity of vaginal microbes, a decrease in the relative abundance of cyanobacteria and Lactobacillus, and an increase in the relative abundance of dialister, peptonephila and other miscellaneous bacteria. There are characteristic vaginal biomarker in normal women, high risk HPV patients and CC patients. In detail, the biomarker in the normal group was varibaculum, the biomarker in the high-risk HPV group was saccharopolyspora, the biomarker of the CC group was the Proteobacteria, Corynebacterium, Coprococcus, Peptococcus and Ruminococcus.

**Conclusions:**

The study indicated that the compositions of vaginal microbes in different cervical lesions is different. The vaginal microbial composition has a certain diagnostic effect on healthy women, patients with high-risk HPV infection and cervical lesions. These microbes may serve as potential biomarkers for CC. It also provided an effective way for the treatment of HPV infections and cervical lesions.

## 1 Background

Cervical cancer (CC) is one of the most common malignancies in women which associated with HPV infection in different regions and populations (Crow, 2012). HPV infection is a common female reproductive tract disease, 70% of women may be infected with this disease in their lifetime (Xia et al., 2023), but so far there is still a lack of effective therapy. How to effectively control the replication of HPV virus has become a challenge for clinicians.

The human microbiota is considered to be the “second human genome” (Integrative HMP (iHMP) Research Network Consortium, 2019). As an important part of microbiota, vaginal microbiome plays a vital role in preventing various infectious diseases of the reproductive tract. Recent studies have shown that there is a relatively relationship between vaginal microbiome, HPV infection and cervical lesions (Chao et al., 2019).

The vaginal microecology is a complex and dynamic system, mainly composed of related anatomical structures, human immunity, internal and external environments, and vaginal microbiome. The normal vaginal micro-ecosystem is parasitized by a variety of bacteria such as lactobacillus, streptococcus, and a various microorganisms such as mycoplasma and viruses, and jointly resist the invasion of external pathogenic bacteria (Liang et al., 2019). When the vaginal microecological balance is broken, it will cause a decrease in the number of lactobacilli, an increase in the number of other miscellaneous bacteria, anaerobic bacteria, and changes in vaginal microbial diversity (Borgdorff et al., 2016), which may lead to a series of reproductive tract diseases. A meta-analysis based on related articles published from 2003 to 2017 showed that among sexually active women, those with vaginal microecological imbalance are more likely to develop persistent HPV infections and increase the risk of cervical intraepithelial neoplasia (CIN) (Brusselaers et al., 2019). Conversely, patients with HPV infection and cervical lesions are also more likely to develop vaginal infectious diseases. The essence of HPV infection is an infectious disease of the reproductive tract. In addition to symptomatic treatment of pathogenic bacteria, attention should be paid to the microecological balance. Therefore, restoring the balance of micro-ecology may be another important way to prevent and treat CC in the future. V4 region is one of the 9 variable regions of 16SrRNA which has a moderate length and high coverage of bacteria. It has been widely used in microbial sequencing and analysis in recent years (Caporaso et al., 2012). In this study we used the illumina high-throughput gene sequencing technology to determine the V4 region of 16SrRNA and observed the vaginal microbial composition in different cervical lesions. The purpose is to explore the causes of HPV infection and cervical lesions, which will provide relevant theoretical basis for the development of vaginal microecological therapy and microecological preparations, and provide new methods for the diagnosis and treatment of HPV infections and cervical lesions.

## 2 Materials and methods

### 2.1 Patients

This study was approved by the Ethics Committee of Suzhou Sci-tech City Hospital. According to the Declaration of Helsinki, written informed consent was obtained from all patients.

All patients were screened strictly according to the following criteria: (1) Having a history of sexual life; (2) Age 20-65 years old; (3) Race is a Han nationality; (4) No history of sexual life, vaginal treatment and flushing 3 days before sampling; (5) No history of antibiotic use within 2 weeks before sampling; (6) No history of radiotherapy and chemotherapy; (7) No history of cervical and vaginal surgery; (8) No sexually transmitted diseases; (9) No abnormal vaginal bleeding or vaginal prolapse and other vaginal diseases; (10) No long-term use of sex hormones, immunosuppressants and steroids; (11) No immune diseases, other malignant tumors and mental diseases; (12) Not during menstruation, pregnancy or lactation. If any criterion is not met, it cannot be included in our study.

125 patients were enrolled in our study who underwent HPV, Thinprep cytologic test (TCT), and colposcopy in the Second Hospital of Dalian Medical University and Suzhou Sci-tech City Hospital from June 2020 to December 2020. HPV typing was performed by PCR and reverse point hybridization which can detect 28 HPV types, including high-risk HPV: HPV16, 18, 31, 33, 35, 39, 45, 59, 52, 53, 56, 58, 59, and 66, common low-risk HPV: HPV68, CP8304, 6, 11, 43, 73, 82, 26, 40, 42, 44, 54, 61, and 83. According to the HPV and TCT results, they were divided into the normal group (n=27, N: HPV and TCT are negative) and the high-risk HPV group (n=40, H: high-risk HPV positive for at least 1 year, TCT negative). Based on postoperative pathological results, patients with shunt colposcopy, colposcopy biopsy or cervical conization were divided into CIN group (n=40, C: CIN2 or 3) and CC group (n=18, Ca: cervical cancer).

The types of HPV infection, age, menopausal history, pregnancy, birth history, and contraceptive methods in each group of patients were analyzed. Using 16SrRNA-V4 region gene amplification technology and Illlumina high-throughput sequencing technology to detect vaginal secretions retained in the GUHE Flora Storage buffer (Zhejiang Hangzhou Equipment Preparation 20190682, GUHE Laboratories, Hangzhou, China), the composition of the vaginal microbiota in each sample is verified. Using bioinformatics analysis methods to analyze the composition of the microbes, diversity of vaginal microbes, microbial markers and microbial predictive ability in each group.

### 2.2 Sampling method

The patients were maintained the lithotomy position, a sterile vaginal speculum without lubricant was used to gently and fully expose the cervix, rotate the sterile cotton swab on the upper 1/3 section of the vagina and the lower section of the vagina for 10-15 seconds, put the cotton swab with vaginal secretion into the storage solution (Zhejiang Hangzhou equipment preparation 20190682), stir and rinse for 30 seconds, discard the cotton swab and mix the storage solution, the specimen bottles were stored in the refrigerator at - 20°C.

### 2.3 DNA extraction

DNA samples from vaginal secretions were extracted using DNA isolation kit (GUHE Laboratories, Hangzhou, China). Agarose gel electrophoresis and Nano Drop ND-1000 spectrophotometer (Thermo Fisher Scientific, Waltham, MA, USA) were used to measure and detect DNA.

### 2.4 16SrRNA amplicon pyrosequencing

The forward primer 515F(5’-GTGCCAGCMGCCGCGGTAA-3’) and the reverse primer 806R(5’-GGACTACHVGGGTWTCTAAT-3’) were used for PCR amplification of the bacterial 16SrRNA gene V4 region. The PCR components contained Phusion High-Fidelity PCR Master Mix (25 μl), which include DNA Template (10 μl), Forward primer(3 μl), Reverse primer(3 μl), DMSO(3 μl), and ddH2O(6 μl). Thermal cycling includes initial denaturation for 30 s (98°C), 25 cycles of denaturation for 15 s (98°C), annealing for 15 s (58°C), extension for 15 s (72°C), final extension for 60 s (72°C). PCR amplicons were purified and quantified using AgencourtAMPure XP Beads (Beckman Coulter, Indianapolis, IN, USA) and the PicoGreen dsDNA Assay Kit (Invitrogen, Carlsbad, CA, USA). The Illumina NovoSeq6000 platform (GUHE Info technology Co., Ltd, Hangzhou, China) was used for paired-end 2×150 bp sequencing.

### 2.5 Sequence analysis

Sequencing data were processed by Quantitative Insights Into Microbial Ecology (QIIME, v1.9.1) (Caporaso et al., 2010). Low-quality sequences were filtered by specific criteria (Chen and Jiang, 2014).Operational taxonomic unit(OTU) was selected by Vsearch v2.4.4 (Rognes et al., 2016) and classified by VSEARCH to search representative sequences in the SILVA132 database (Quast et al., 2013). To prevent environmental contamination, all samples in the project were processed side by side using the same batches of reagents, at the same time, we have added a sample with a control check and a native sample of a standard strain. Decontam was used to remove contamination sequences.

### 2.6 Bioinformatics and statistical analysis

Sequence data were analyzed by R packages (v3.2.0) and QIIME. The OTU table was used for the calculation of OTU-level alpha diversity and generation of OTU-level ranked abundance curves. Taxa abundance at the phylum, class, order, family, genus and species levels were compared among samples or groups by Kruskal test from R stats package. Kruskal.test and Wilcox.testwere were used to analyze the alpha diversity of each group. Anosim test was used to determine whether the grouping was meaningful. Linear discriminant analysis was used for effect size (LEfSe) to detect significantly different taxa across groups (Segata et al., 2011).

### 2.7 Data analysis

SPSS 21.0 (SPSS, Chicago, Illinois, USA) was used for statistical analysis of clinical data. Continuous variables and categorical variables was analyzed by the t-test and χ2 test or Fisher’s test separately. P values <0.05 were considered to be statistically significant.

## 3 Results

### 3.1 Clinical data

97 of the 125 patients, were infected with HPV. In those 97 HPV patients, the most HPV subtype is type 16, accounting for 47.4% (n=46), followed by type 52 (22.7%) (n=22), type 58 (16.5%) (n=16) and type 18 (12.4%) (n=12) respectively. In the high-risk HPV group, HPV16 and/or 18 infections accounted for 52.5% (n=21). In the CIN group, HPV16 and/or 18 infections accounted for 50% (n=20), and 1 case without HPV infection, accounting for 2.5%. In the CC group, HPV16 and/or 18 infection accounted for 94.4% (n=17). The ages of the enrolled patients were 21 to 65 years old. There were no statistical differences in general clinical data among the groups including age, menopausal history, fertility status, contraceptive methods and BMI (**Table 1**).

**Table 1:** Clinical data.

characteristics	N (n=27)	H (n=40)	C (n=40)	Ca (n=18)	χ^2^ value	P value
age					7.15	0.067
≤50	24	32	31	10		
>50	3	8	9	8		
menopausal history					6.94	0.074
premenopausal	24	28	31	10		
postmenopausal	3	12	9	8		
fertility status					5.88	0.105*
had not given birth	6	6	3	0		
had given birth	21	34	37	18		
contraceptive methods					14.5	0.192*
Non-contraception	9	20	13	12		
safe period	0	1	0	0		
condom	13	16	17	4		
Intrauterine device	5	3	9	2		
Sterilization	0	0	1	0		
BMI					3.354	0.773*
≤18.5	7	11	8	7		
18.5-25	15	22	27	9		
≥25	5	7	5	2		

P value is calculated using χ2 and Fishers test (*).

### 3.2 Microbial composition

**Figure 1:**
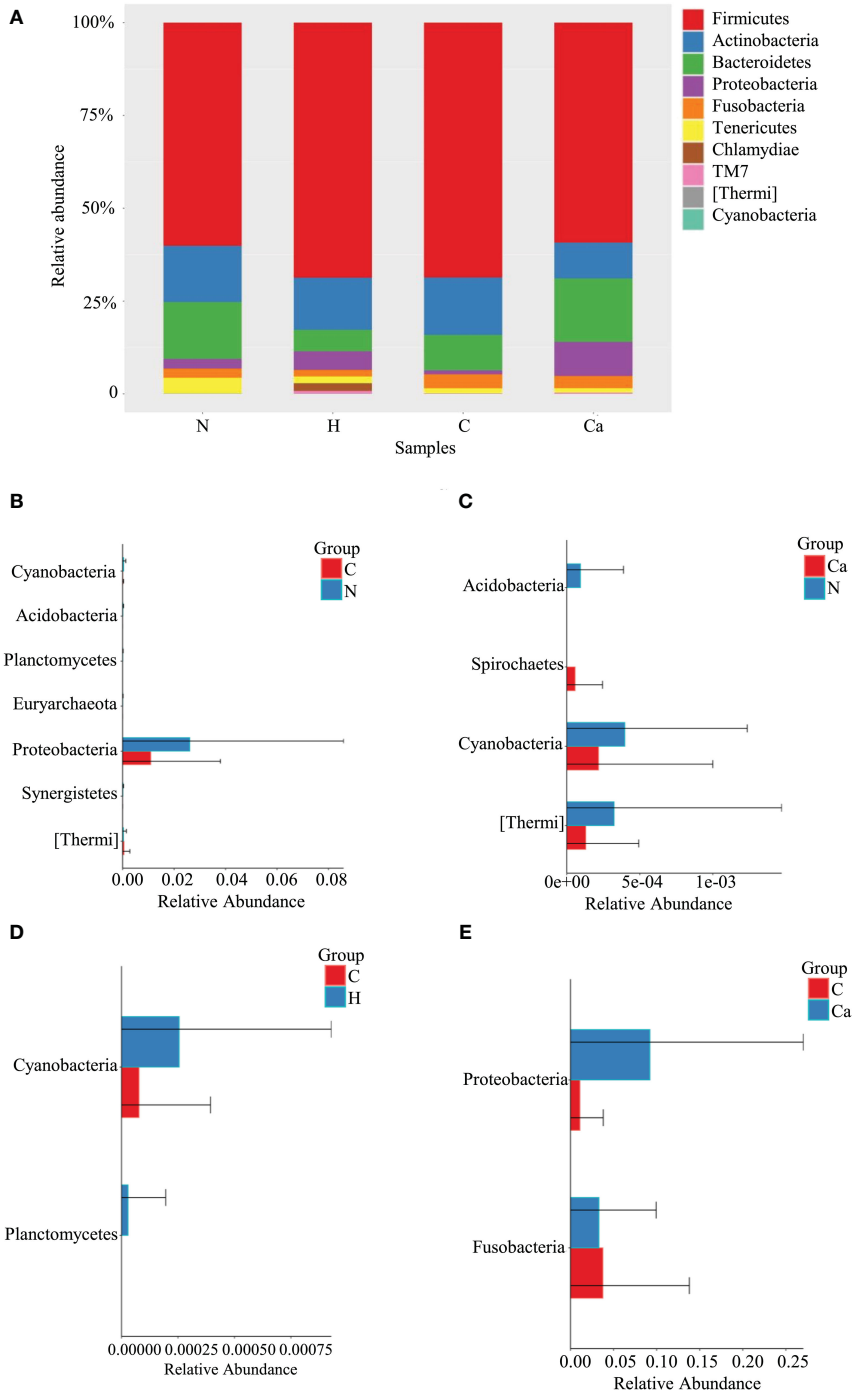
Comparative analysis of the relative abundance of phyla in four groups. **(A)** Bar chart of relative abundance of top 10 phyla in four groups. **(B)** Histogram of differential phylum between Group N and Group C. **(C)** Histogram of differential phylum between Group N and Group Ca. **(D)** Histogram of differential phylum between Group H and Group C. **(E)** Histogram of differential phylum between Group C and Group Ca.

The Relative abundance of phyla in four groups was shown in **Figure 1**. The Phylum Firmicutes occupied the highest proportion. The relative abundances of Firmicutes in the normal group, high-risk HPV group, CIN group and CC group were 60.1%, 68.7%, 68.6%, and 59.2%, respectively and there was no significant difference among the groups. The other top 10 phyla in vagina are Actinobacteria, Bacteroides, Proteobacteria, Fusobacteria, Tenericutes, Chlamydiae, TM7, Thermi, and Cyanobacteria (**Figure 1A**). The Wilcoxon test results showed that compared to the normal group, the relative abundance of Cyanobacteria and Proteobacteria in the CIN group was significantly decreased, and that of Thermi was significantly increased, while that of Cyanobacteria and Thermi in the CC group was significantly decreased. Compared with the CIN group, the relative abundance of Fusobacteria in the CC group was significantly decreased while that of Proteobacteria was significantly increased, In addition, the relative abundance of Cyanobacteria in the CIN group was significantly lower than that of high-risk HPV group (**Figures 1B–E**).

**Figure 2:**
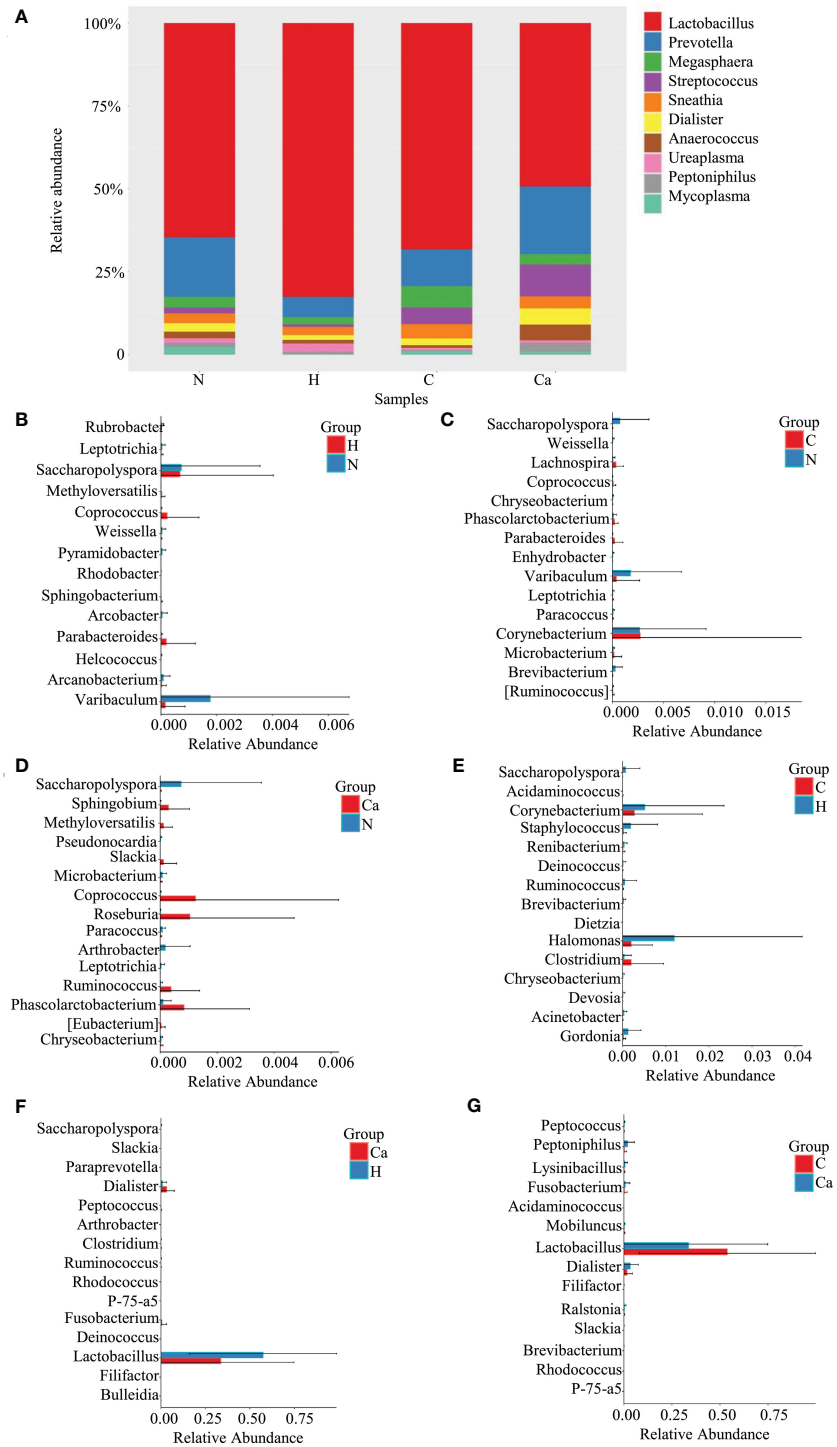
Comparative analysis of the relative abundance of genera in four groups. **(A)** Bar chart of relative abundance of top 10 genera in four groups. **(B)** Histogram of differential genera between Group N and Group H. **(C)** Histogram of differential genera between Group N and Group C. **(D)** Histogram of differential genera between Group N and Group Ca. **(E)** Histogram of differential genera between Group H and Group C. **(F)** Histogram of differential genera between Group H and Group Ca. **(G)** Histogram of differential genera between Group C and Group Ca.

The Relative abundance of genera in all groups was shown in **Figure 2**. The Lactobacillus occupied the highest proportion. The relative abundances in the normal group, high-risk HPV group, CIN group, and CC group were 48.9%, 57.4%, 53.8%, 33.6% and the remaining top 10 bacterial genera included Prevotella, Megasphaera, Streptococcus, Sneathia, Dialister, Anaerococcus, Ureaplasma, Peptoniphilus, and Mycoplasma (**Figure 2A**). The Wilcoxon test results showed that there were significant differences in bacterial genera among groups. For the top 10 bacterial genera, there are significant differences between the high-risk HPV group and the CC group or between the CIN group and the CC group. Compared with the high-risk HPV group, the relative abundance of Lactobacillus in the CC group was significantly reduced while that of Dialister was significantly increased. Compared with the CIN group, the relative abundance of Lactobacillus in the CC group was also significant reduced, while that of Dialister and Peptoniphiluswere significantly increased (**Figures 2B–G**).

### 3.3 Microbial Alpha diversity

**Figure 3:**
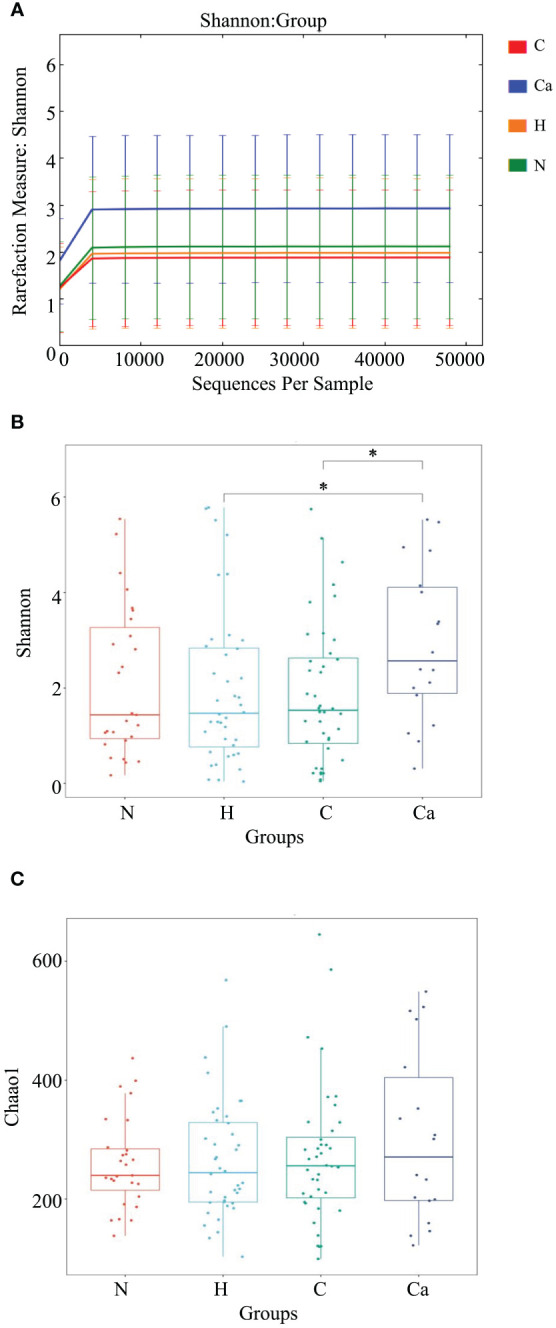
The microbial Alpha diversity analysis in different groups. **(A)** Shannon-Winner curve in different groups. **(B)** Shannon index box chart for each group. **(C)** Chao1 index box chart for each group. (*P<0.05).

First, we described the Alpha diversity of the bacterial community by measuring Chao1 index and Shannon index. Second, we created the rarefaction curves to test the sequencing depth and the result showed that the amount of sampling is reasonable (**Figure 3A**). We found the Shannon index of the CC group was significantly higher than that of the HPV group (P=0.034) and the CIN group (P=0.019) (**Figure 3B**). But there was no significant difference of the Chao1 index between groups (P>0.05) (**Figure 3C**).

### 3.4 Biomarkers

**Figure 4:**
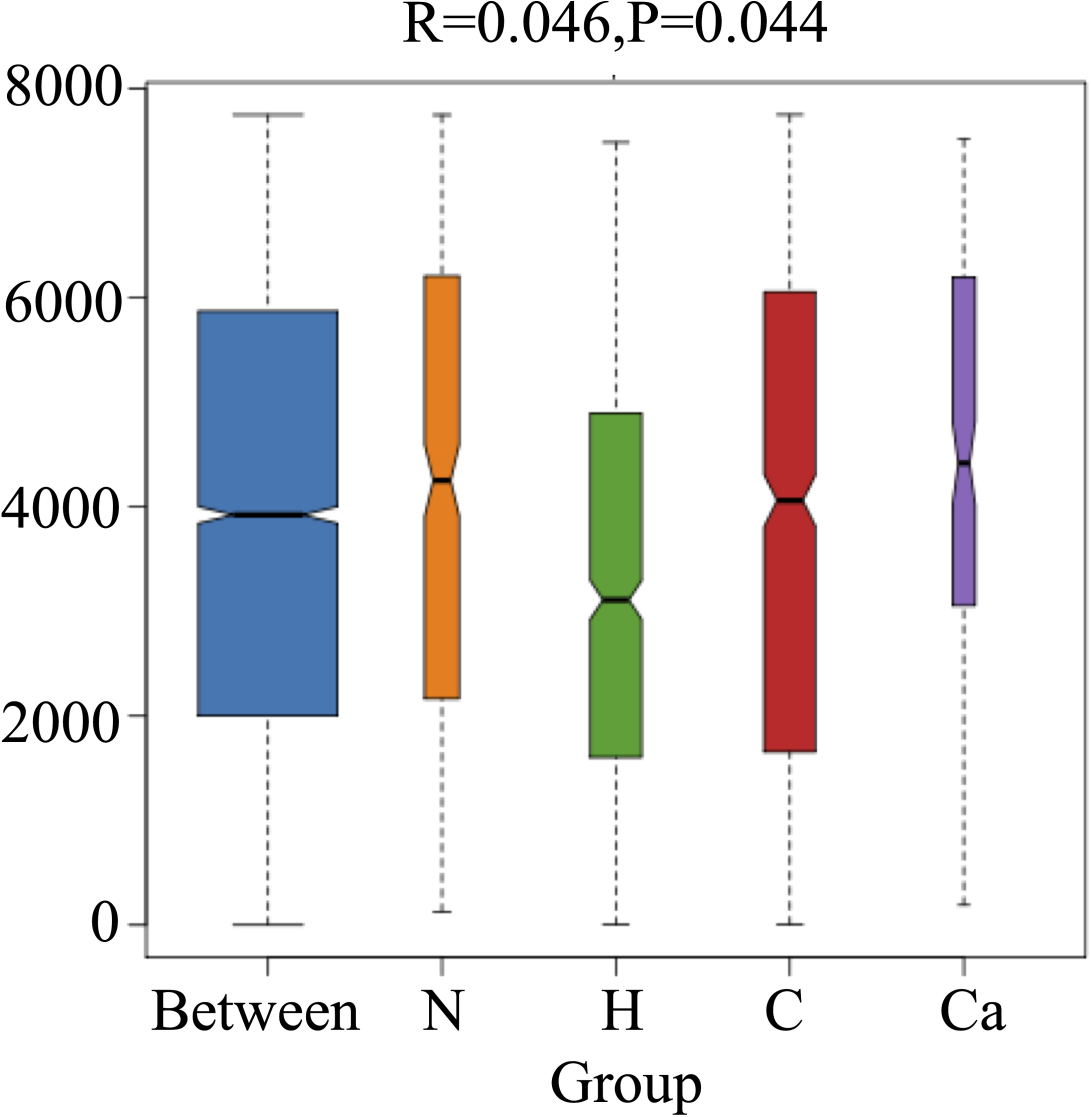
Anosim test box diagram of each group.

**Figure 5:**
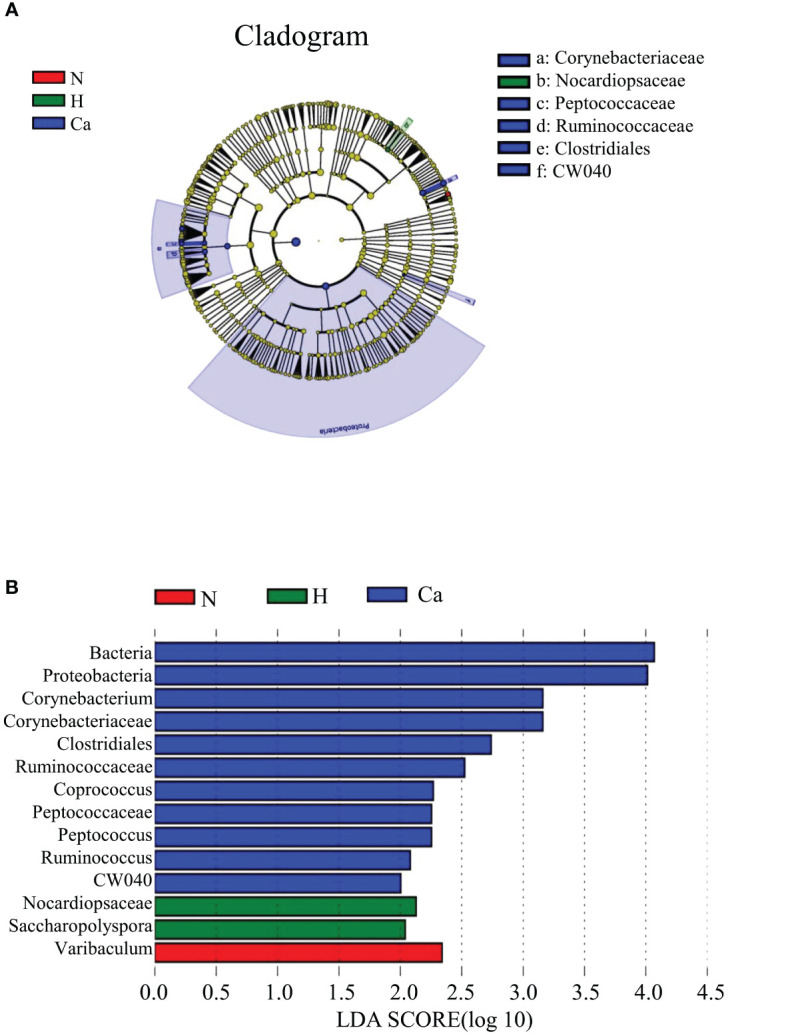
Biomarkers of each group. **(A)** The evolutionary branch diagram of microorganism with significant differences in each group. **(B)** LDA value histogram of microorganism with significant differences in each group.

In order to fully determine the role of vaginal microbiota and the differences in microbial between the groups, we performed the Anosim test. The results showed that there were significant differences between the groups(P=0.044) (**Figure 4**). Furthermore, the composition of vaginal microbes in each group was analyzed by LEfSe, and **Figure 5** showed the clade diagram and bar graph of LDA value (only microbes with LDA>2).In this way, biomarkers (species with significant differences between groups) were screened between groups. The difference markers between groups selected by LEfSe are the dominant species, and these markers are distinguished by specific significant differences between this group and other groups. At the phylum level, the biomarker of the CC group was the Proteobacteria, and at the genus level, the biomarkers of the CC group were Corynebacterium (LDA=3.16), Coprococcus (LDA=2.27), Peptococcus (LDA=2.25) and Ruminococcus (LDA=2.08), the biomarker in the high-risk HPV group was saccharopolyspora (LDA=2.04), the biomarker in the normal group was varibaculum (LDA=2.34), and no biomarker have been found in the CIN group (**Figure 5**).

### 3.5 Microbial prediction ability

**Figure 6:**
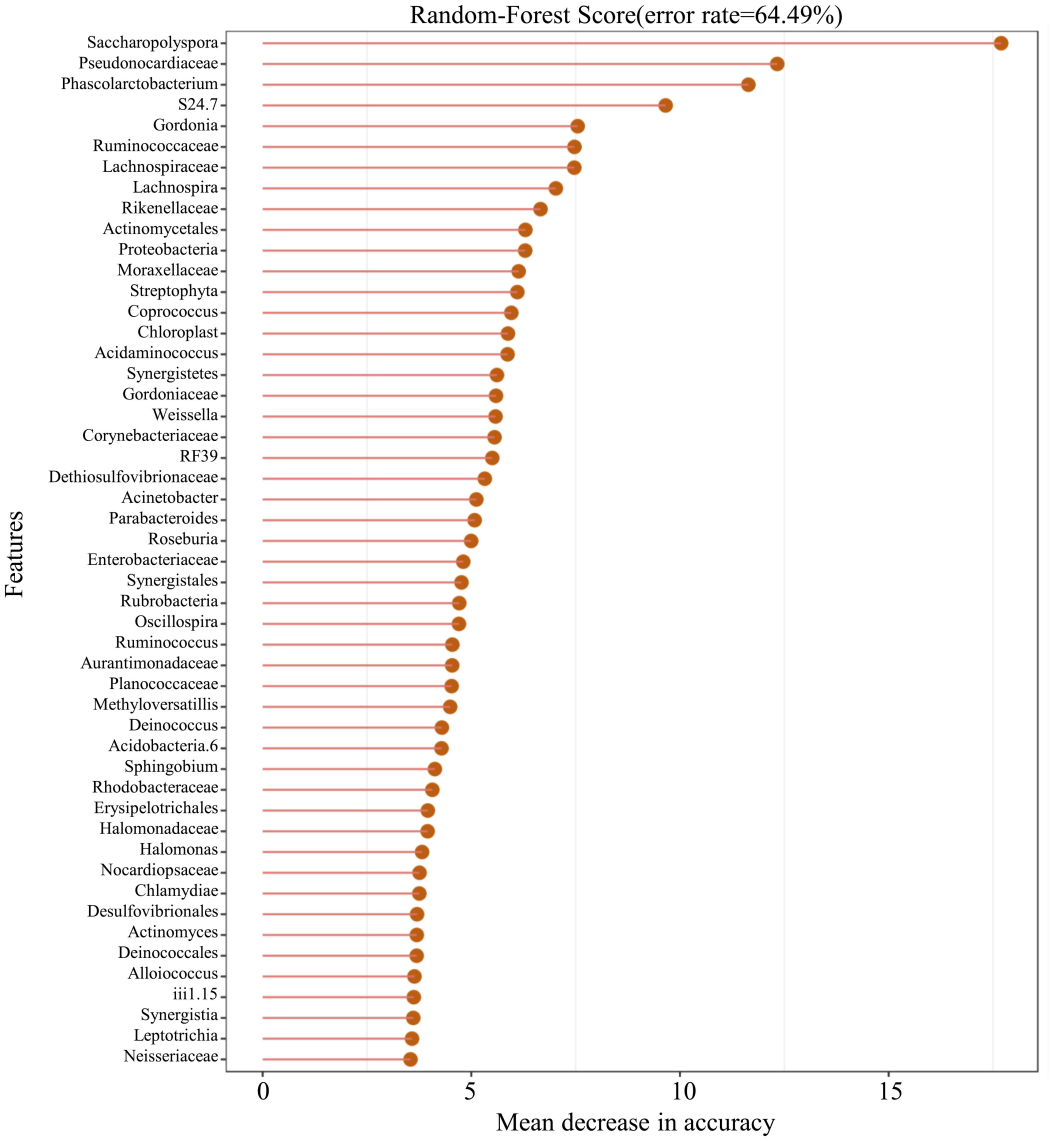
Random-forest graph of top 50 different bacterial communities between each group.

**Figure 7:**
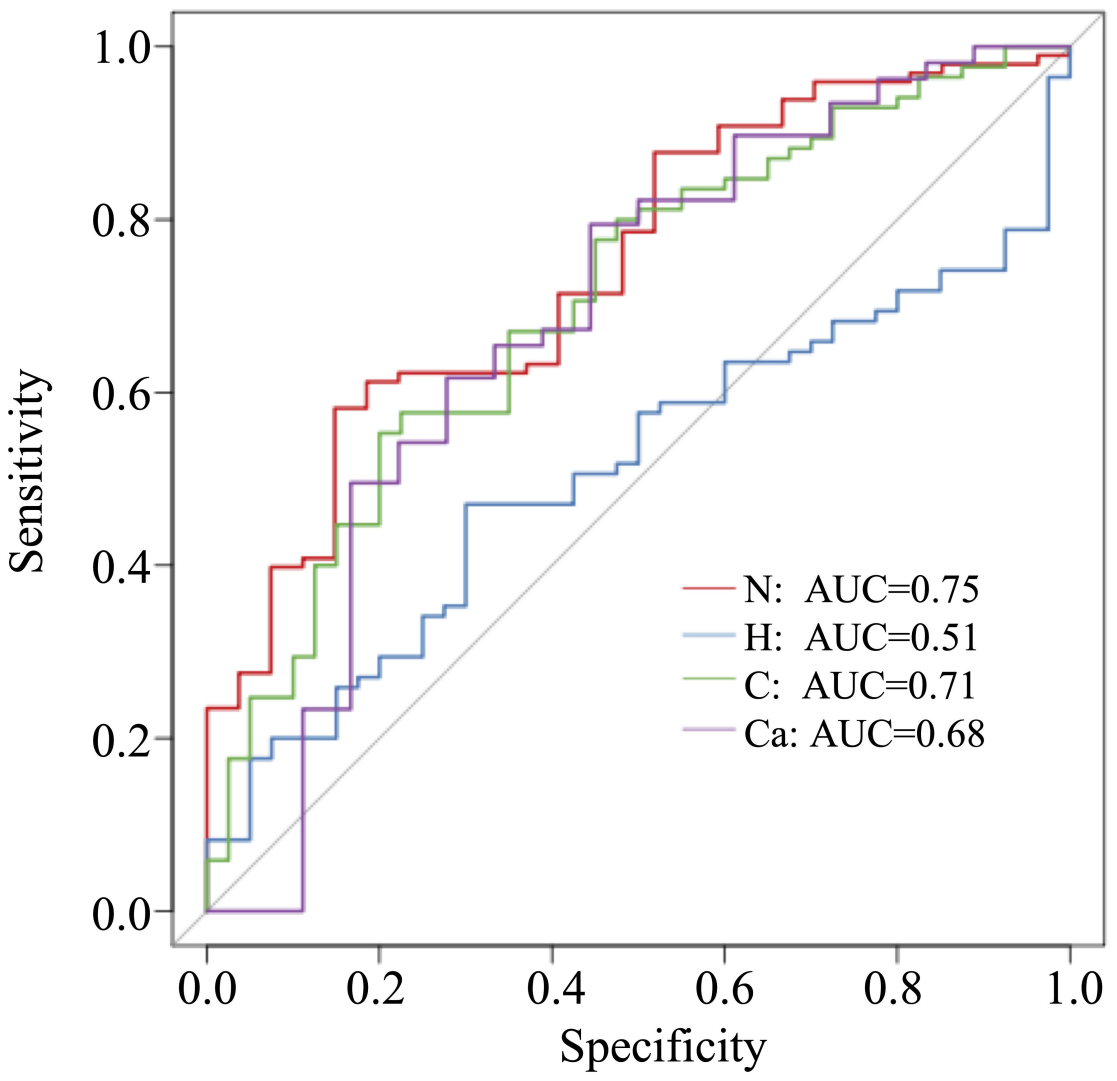
ROC curve of each group.

In this study, 50 different bacterial communities were screened, a random forest graph was made (**Figure 6**), and an ROC curve was plotted (**Figure 7**). The results showed that the vaginal microbial composition had a predictive effect on each group. The prediction accuracy of HPV group (AUC=0.51) and CC group (AUC=0.68) was lower than that of the normal group (AUC=0.75) and CIN group (AUC=0.71).

## 4 Discussion

The female reproductive tract is a channel that communicates with the outside world which is easily invaded by external microorganisms, leading to HPV infections and other infectious diseases. The infection of high-risk HPV subtypes may lead to the CC (Yang et al., 2020). Consistent with the previous study, this study showed that more than 90% of patients with CIN or cervical cancer are infected with high-risk HPV. Among all HPV subtypes, types 16 and 18 are considered to have the highest carcinogenic effects (de Sanjosé et al., 2007) (Torre et al., 2015). However, most HPV infections subside within a short period of time, and only a few patients will gradually cause CC after prolonged infection. It is known that the vaginal microecology has played an important role in the process of HPV infections-induced cervical lesions (Kovachev, 2020). Vaginal microecology is affected by the factors such as age, menstruation, medication, vaginal lavage, contraceptive methods and other factors. It is a dynamic equilibrium system formed by the mutual restriction and coordination of microorganisms and the environment in the host (Mitra et al., 2016). If the vaginal microecological balance is broken and the immune system is compromised, foreign microorganisms will invade the reproductive system and reduce the immune system’s clearance rate of HPV and other pathogens (Chehoud et al., 2017).

Firmicutes is the dominant phyla and Lactobacillus is the dominant genera for normal vaginal microbiome. Similar to previous studies (Walther-António et al., 2016; Chao et al., 2019; Norenhag et al., 2020; So et al., 2020; Xie et al., 2020), our study found that Firmicutes did not differ significantly between the groups. Compared with the cervical lesions group, Lactobacillus were significantly reduced in the CC group, while the relative abundance of Dialister and Peptoniphilus was significantly increased. It has been suggested that pathogenic mechanism of Peptoniphilus may be related to its strong adhesion to the vaginal epithelium and the formation of biofilms (Patterson et al., 2010), while the pathogenic mechanism of Lactobacillus may vary with different strains (Ravel et al., 2011; Teka et al., 2023). The research on Proteobacteria is inconclusive (Chao et al., 2019; Cheng et al., 2020). Recent study has found that Proteobacteria in colon cancer are positively correlated with tumor burden (Lee et al., 2021). Our study also showed that Proteobacteria in CC patients has significantly increased compared with CIN patients. It indicated that Proteobacteria may be involved in tumor progression. Fusobacteria is another one of the common bacterial phyla in the vagina. Harrandahet al. found that Fusobacteria potentially enhanced the invasiveness and survival rate of cancer cells in the oral cavity (Harrandah et al., 2020), but our results demonstrated that the relative abundance of Fusobacteria has decreased significantly in the development of cervical lesions, The reasons for this difference may due to the selection of different cases, different geographic region and microenvironment. Therefore, it needs to be further explored.

There are few studies on the distribution and function of cyanobacteria in human microecology all over the world. Previous studies have shown that cyanobacteria are one of the common intestinal microorganisms of small intestinal adenocarcinoma (Pan et al., 2021). Cyanobacteria are also described in the breast. Some studies show that the relative abundance of cyanobacteria in Chinese women is significantly higher than that in Slovak women and it indicates that cyanobacteria are related to the geographical area. The relative abundance of cyanobacteria in primary breast cancer is lower than that of healthy women in China (Hadzega et al., 2021) and this result similar to ours. Although its mechanism is unclear, it suggests that cyanobacteria may play an inhibitory role in tumor progression. However, there are few studies on the physiological role and mechanism of cyanobacteria in the vaginal. Our findings may lay a foundation for future research. Previous studies have shown that thermi may be related to HPV16 (Huang et al., 2018). Other studies have shown that the relative abundance of thermi is higher in advanced lung cancer than in early lung cancer (Yu et al., 2016). Our study also suggests that the relative abundance of thermi in CIN group is high. It indicates that thermi may be related to tumors development. However, the specific mechanism remains to be further explored.

In recent years, the research on the diversity of the vaginal microbiome has gradually become a hot topic, and Alpha diversity is the main indicator to measure the diversity and richness of microbial communities. The diversity index is mainly used to measure the heterogeneity of the community, and the richness is mainly used to measure the number of species in a single sample. A study on the change of vaginal microecological under different cervical lesions showed that patients with normal cervix had a higher vaginal microbiome richness than patients with cervical lesions, but the difference was not statistically significant (Wu et al., 2020). Another study has also shown that there is no significant difference in the abundance and diversity of vaginal bacteria under different cervical lesions, but HPV infection significantly increases the abundance and diversity of vaginal bacteria (Chen et al., 2020). Mitra et al. found that following the degree of cervical lesions increases, the richness and diversity of vaginal bacteria also increase (Teka et al., 2023). In current study, the Shannon index was used to estimate the microbial diversity, and the Chao1 index was used to estimate the microbial richness. The results showed that there is no significant difference in microbial richness but the diversity of vaginal microbiome in patients with CC was significantly higher than that in patients with high-risk HPV infection and CIN patients. These different results in various studies on the diversity of vaginal microbiome may be related to different geographic regions, races, sample numbers, and sample collection techniques (Kovachev, 2020). A study involving almost all ethnic females showed that the risk of increased microbiota diversity and vaginal microecological imbalance in Caribbean African women is four times than that of European/Caucasian and African women (Mortaki et al., 2020), indicating that race is a key factor affecting the vaginal bacterial community. All the research objects of this study are from the Han nationality, it only represent the characteristics of vaginal microorganisms of the Han nationality. Based on the above studies, a worldwide multi-center study may be quite necessary.

It has been believed that there are characteristic biomarkers in patients with HPV infection and different degrees of cervical lesions, the biomarkers derived from the results of various studies are different. In recent years, studies found that Delftia and Gardnerella may be biomarkers of cervical precancerous lesions (Usyk et al., 2020; Wu et al., 2020) and bacteria such as Gardnerella vaginalis, Gardnerella, Peptostreptococcusanaerobius, Mobiluncuscurtisii, Prevotella genus and Fusobacterium nucleatum may be characteristic biomarkers of HPV16-positive patients (Yang et al., 2020). This study used LEfSe analysis to explore the characteristic biomarkers and showed that Proteobacteria, Corynebacterium, Coprococcus, Peptococcus and Ruminococcus may be used as biomarkers for CC patients. The biomarker for high-risk HPV patients is Saccharopolyspora, and the biomarker for normal patients is varibaculum. These characteristic bacterial genera are not only related to the degree of different cervical lesions, but may also be related to certain reproductive tract infections. Previous studies have shown that Proteobacteria (Wei et al., 2023), Corynebacterium (Wang et al., 2014) and Peptococcus (Lefèvre et al., 1985) are related to bacterial vaginosis. The relationship between CC and bacterial vaginosis is also worthy of further study. Varibaculum is a kind of actinomycete and previous studies have shown that its specific strain may exist in the intestine of premature infants (Brown et al., 2013). And a data searched in HMP showed that Varibaculum in healthy human microecology were mainly distributed in the vagina (Integrative HMP (iHMP) Research Network Consortium, 2019) and it is consistent with our results. Recently, a new strain of varibaculum has been extracted from the vagina of healthy Senegalese women (Fall et al., 2019). Therefore, the distribution and mechanism of varibaculum and its subordinate species may be a new research field of vaginal microecology. The composition of the vaginal microbiome is different between patients with CIN1 and CIN2, and this different composition has extremely high accuracy in predicting the severity of CIN (Lee et al., 2020). Our results also demonstrated that the composition of vaginal microbes may predict high-risk HPV infection and cervical lesions.

## 5 Conclusion

This study found that the vaginal microecology is different under various pathological conditions of the cervix, but no difference in the richness of vaginal microbes. The diversity of vaginal species in CC patients is mainly manifested as a reduction in the number of Cyanobacteria and Lactobacillus, accompanied by an increase in the number of Dialister, Peptoniphilus and other miscellaneous bacteria. Different cervical lesions have specific biomarkers, so the composition of the vaginal microecological flora has a diagnostic effect on cervical lesions. These results may provide new evidences for the diagnosis of HPV infection and cervical lesions. Due to the changes in the vaginal microecology under cervical lesions, restoring the balance of vaginal microecology may be an effective way for the treatment of HPV infection and cervical lesions.

## Data Availability

The raw data used to support the findings of this study are provided in the following links: https://www.jianguoyun.com/p/DdRG93UQ_svSDBjQidkFIAA.
